# Unconventional Secretion of Heat Shock Proteins in Cancer

**DOI:** 10.3390/ijms18050946

**Published:** 2017-04-29

**Authors:** Tiago Góss Santos, Vilma Regina Martins, Glaucia Noeli Maroso Hajj

**Affiliations:** International Research Center, A.C. Camargo Cancer Center, São Paulo 01508-010, Brazil; tsantos@cipe.accamargo.org.br (T.G.S.); vmartins@cipe.accamargo.org.br (V.R.M.)

**Keywords:** heat shock proteins, cancer, unconventional secretion, exosomes

## Abstract

Heat shock proteins (HSPs) are abundant cellular proteins involved with protein homeostasis. They have both constitutive and inducible isoforms, whose expression levels are further increased by stress conditions, such as temperature elevation, reduced oxygen levels, infection, inflammation and exposure to toxic substances. In these situations, HSPs exert a pivotal role in offering protection, preventing cell death and promoting cell recovery. Although the majority of HSPs functions are exerted in the cytoplasm and organelles, several lines of evidence reveal that HSPs are able to induce cell responses in the extracellular milieu. HSPs do not possess secretion signal peptides, and their secretion was subject to widespread skepticism until the demonstration of the role of unconventional secretion forms such as exosomes. Secretion of HSPs may confer immune system modulation and be a cell-to-cell mediated form of increasing stress resistance. Thus, there is a wide potential for secreted HSPs in resistance of cancer therapy and in the development new therapeutic strategies.

## 1. Heat Shock Protein Functions and Families

Heat shock proteins (HSPs) were identified initially as proteins necessary for stress responses, such as temperature elevation and other proteotoxic stresses, preventing the damage of cellular structures, thus protecting essential cellular functions [1]. However, it was also envisioned that HSP families have members that are constitutively expressed [2]. The major functions of HSPs are to assist protein folding and prevent the formation of nonspecific protein aggregates [3].

There are five large and ubiquitous HSP families [4]: HSP70 superfamily (that includes HSP70 and HSP100 proteins) contains 17 genes, DNAJ family (also known as HSP40 family) contains 49 genes, small heat shock proteins (that includes HSP27) contains 11 genes, the HSPC family (also known as HSP90 family) contains five genes and the chaperonin family (that includes HSP60) contains 14 genes [5]. The nomenclature for HSPs has been diverse in the literature, which generates a lot of confusion. Table 1 summarizes the HUGO Gene nomenclature and most frequent names in the literature for the HSPs cited in this review.

HSPs function as chaperones, facilitating protein folding of client proteins. Thus, they act in the co- or post-translational folding of newly synthetized proteins and on the remodeling of misfolded proteins that can be caused by heat shock and other stress conditions. They can even aid the clearance of protein aggregates and are essential for the activity of many proteins. HSPs are thus found in most cellular compartments, such as cytoplasm, endoplasmic reticulum (ER) and mitochondria [30].

From the almost one hundred known HSPs, at least 13 were found extracellularly in many different biological models, from cell lines to whole organisms, in both physiological and pathological conditions (Table 1). Unlike intracellular HSPs, the functions of secreted forms are still under debate; however, most articles in the literature suggest that they may represent a signal for immune system modulation.

## 2. The Discovery of Chaperone Secretion

The surprising observation that HSPs can be actively secreted from cells was reported almost 30 years ago, when investigators discovered that cultured cells released HSPH1 and HSPA8 after a short heat shock stress [31]. HSPA1A was also found in the extracellular membrane of maturing reticulocytes in association with the transferrin receptor [32]. In spite of the initial skepticism regarding the secretion of proteins that are predominantly cytoplasmic, many reports were able to replicate these findings, providing evidence that HSP release is an active mechanism rather than a non-specific event induced by cell lysis. Further reports also demonstrated in vivo extracellular HSPs, with the observation of HSPD1 and HSPA1A in normal human blood circulation [33,34].

Tumor cells were among the first systems in which extracellular HSPs were documented. HSP90AA1 and HSPA1A were detected at the surface of tumor cell lines (microcitoma, lung carcinomas, melanomas, Ewing’s sarcomas, osteosarcomas and hepatomas) [35,36]. In addition, early reports also suggested that cell surface expression of HSPA1A in colon carcinoma cells (CX2) would increase cell recognition by the immune system, thus representing an anti-tumor effect of extracellular HSPs [37,38].

Extracellular HSPs were also regarded as potential cytokines. For example, HSPA1A was pointed to have a role in the stimulation of macrophages to secrete proinflammatory cytokines and to induce the expression of antigen-presenting molecules on dendritic cells [39,40].

## 3. DAMP vs. DAMPER—Dual Role of Extracellular HSPs?

HSPs are frequently associated with damage-associated molecular patterns (DAMPs), a class of self-danger signals released by stressed cells that elicit immune responses [41]. This term was conceived in analogy to the term PAMP (pathogen-associated molecular patterns), which is a class of molecular signals from pathogens that activate innate immune system [42]. DAMP signals are also known as alarmins and comprise different groups of intracellular components (such as the DNA-binding protein HMGB1, S-100 family proteins, nucleosomes, ATP, uric acid and antibacterial peptides) that are released during necrotic (not apoptotic) cell death and stimulates the immune system [42]. Kono and Rock revised the criteria for the classification of DAMPs [43], which are: “(1) DAMP should be active as a highly purified molecule; (2) it is important to show that its biological activity is not owing to contamination with microbial molecules (PAMPs); (3) the DAMP should be active at concentrations that are actually present in pathophysiological situations; and (4) selectively eliminating or inactivating the DAMP should ideally inhibit the biological activity of dead cells in in vitro and in vivo assays.”

For a long time, it was a consensus in the scientific community that HSPs could act as DAMPs, due to their role in cell stress responses and for their release to extracellular milieu during stressful conditions. However, recent proposals argue against this nomination [44], since HSPs do not fulfill the criteria properly. For example, the mechanisms that promote HSP secretion are active even in physiological situations. In addition, many experiments that consider HSPs as immunostimulatory molecules were done with recombinant protein systems, which have the bias of putative Lipopolysaccharide (LPS) contamination from bacteria [45].

One example includes HSPH1 and HYOU1, which were first described to have a role in the activity of scavenger receptors on macrophages and dendritic cells [6,9]. On the contrary, further work demonstrated inhibittory effects of extracellular HSPH1 and HYOU1 upon macrophage differentiation, favoring a pro-tumor phenotype [10].

Thus, in opposition to the denomination of HSPs as DAMPs, the term DAMPER has been suggested, which means a class a molecules that would reduce the activity of the innate immune system [44,45]. Additional evidence for this immunosuppressive feature for extracellular HSPs were suggested by others [46,47,48,49,50].

Lastly, it seems that the cellular context in which HSPs reach extracellular environment is determinant for its activity. For instance, during stressful conditions that lead to massive necrosis, HSP levels could be elevated by cell lysis, which would trigger an immunostimulatory phenotype. However, during physiological conditions, the cells release HSP by controlled mechanisms that may trigger immunosuppression [49]. The determination of binding receptors for HSPs may offer and answer for the amplitude of the phenomena associated with HSPs in the extracellular milieu.

## 4. Unconventional Mechanisms of HSP Secretion

The mechanisms related to HSP release are highly controversial. The most relevant argument against a specific HSP secretion is the absence of a signal peptide that targets these proteins for classical secretion. Indeed, the secretion HSPA1A and HSPA8 was shown to be resistant to brefeldin A, an inhibitor of classical secretion [51]. However, this observation was challenged by other groups that suggest the participation of endolysosomal compartments in the secretion of HSPA1A, through the inhibition by lysosomotropic compounds (methylamine or ammonium chloride). These studies suggest that the HSP entry into the secretory compartments may be mediated by ABC transporters, in a mechanism similar to that observed for interleukin-1β secretion [52,53]. The secretion of HSPs can also be explained by unconventional mechanisms. For example, inhibition of the lipid raft dynamics using methyl-β-cyclodextrin reduced HSPA1A and HSPA8 release [51,54,55,56]. A second mechanism was suggested with the participation of exosomes in a pathway that involved signaling through the extracellular regulated kinase 1/2 (ERK1/2) and phosphatidylinositol-3 kinase (PI3K) pathways [57,58,59,60,61].

Regardless of the controversial secretion mechanisms, the majority of the literature indicates that HSPA1A secretion is modulated by stress, with an increase in the secretion caused, for example by heat shock or other chemical stresses [54,55,56,57,62]. Interestingly, it was noted that upon heat shock or pharmacological inhibition of phospholipase C with u73122, HSPA1A is translocated from the cytoplasm to secretory-like granules and that its secretion could be blocked by brefeldin A [63,64]. In vivo, this may be responsible for the observed increase in serum HSPA1A under stress conditions such as trauma [65], cardiovascular disease [66], pulmonary edema [67], radiation therapy [68], surgery interventions [69], pathological conditions such as diabetes [70], or even exercise [71,72].

HSPA1A was also observed in the cell membrane. In colon carcinoma cellular models (CX2), hypoxia treatment triggered a co-localization of HSPA1A with phosphatidylserine on the cell surface, which reduced cell viability [73]. HSPA1A in the membrane was also observed in vivo in a variety of human tumors, such as: colorectal, lung, neuronal, and pancreas carcinomas; liver metastases; leukemic blasts [74]; squamous cell carcinomas [75,76]. A direct integration of HSPA1A on the plasma lipid bilayer was suggested as a possible mechanism, supported by the evidence that recombinant HSP can integrate in artificial membranes and create ionic channels [77,78,79,80,81].

Other HSPs were found to be secreted as well, with equal controversies regarding the mechanisms of secretion. Early reports detected HSPD1, a chaperone classically found in the mitochondria, in cell culture supernatants of neuroblastoma and glioblastoma cell lines, with an increase after cell stress [82]. This report suggested a role of classical secretion, which was supported by later findings that shows HSPD1 secretion by endoplasmic reticulum-Golgi pathways [83]. Nevertheless, additional literature in both normal tumor cells lines suggested a mechanism dependent on exosomes [84,85]. Thus, an interesting hypothesis is that there is participation of both mechanisms in HSPD1 secretion. Indeed, reports show that HSPD1 was found by electron microscopy in the membrane of human lung mucoepidermoid (H292) and lung adenocarcinoma (A549) cells and derived exosomes and its secretion was also inhibited by Brefeldin A [86]. In vivo, HSPD1 was found in the plasma of normal subjects [87]; however, it has been observed that circulating HSPD1 was enhanced in patients with borderline hypertension [88]. HSPD1 in the circulation can origin from the anterior pituitary, pancreatic acinar cells [89] and β-cells [90], where HSPD1 localizes in secretory granules. The presence of HSPD1 in the surface of exosomes was observed in large bowel patients, whose levels were reduced after tumor ablative surgery [91].

Other HSPs whose secretion has been related to exosomes are HSP90AA1 [92], HSP1B [93] and HSPB6 [25]. HSP90AA1 was shown to interact with annexin II and tissue plasminogen activator in exosomes to increase plasmin dependent cell motility [92]. HSPB1 is another chaperone secreted by cells in a non-classical pathway [94] associated to exosomes [93]. In vivo, serum HSPB1 was observed increased in pancreatic carcinoma [95], hepatocellular carcinoma [96], breast cancer [97] and gastric adenocarcinoma patients [98].

On the other hand, HSPs may have a role in the mechanism of non-classical secretion. For example, in retinal pigment epithelial cells inhibition of the small heat shock protein CRYAB (αβ-crystallin) by shRNA, reduced exosomal secretion and increased the presence of vacuoles and large vesicles, suggesting an alteration of the endolysosomal traffic associated to exosome formation [99]. αβ-crystallin itself is also secreted by exosomes [100].

Finally, no universal pathway for HSP release has been identified thus far, although there is strong evidence for exosome-mediated secretion. However, the mechanisms of the sorting of HSPs to this kind of vesicles are elusive. One suggested mechanism is related to post-translational modifications that regulate the incorporation of HSP into exosomal cargo. Ubiquitination seems to be the most prominent mechanism [101], but evidence points that SUMOylation, phosphorylation, glycosylation, myristoylation and oxidation are also involved in sorting proteins to multivesicular bodies to be later released to extracellular milieu [102]. Interestingly, exosome formation pathways can be controlled by several oncogenes [103,104,105,106] and tumor suppressor genes [107,108,109], highlighting the importance of exosomes and their cargos to promote oncogenic signals and also to establish therapeutic strategies focused in the endocytic system [110].

## 5. Functions of Extracellular HSPs in Cancer

**Figure 1:**
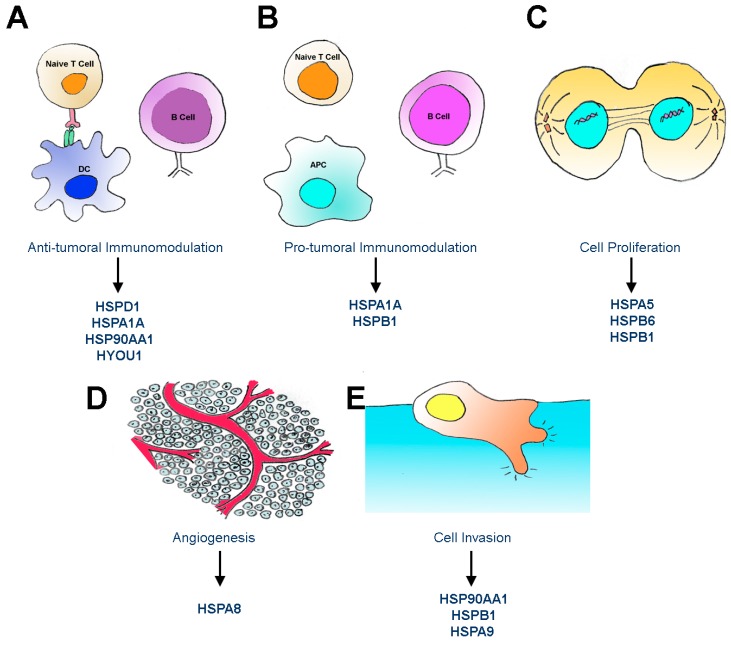
Functions of extracellular HSPs in cancer. Secreted HSPs whose functions were described as (**A**) anti-tumor immunomodulation; (**B**) pro-tumor immunomodulation; (**C**) cell proliferation; (**D**) angiogenesis; (**E**) cell invasion. APC, antigen presenting cell; DC, dendritic cell.

One of the proposed functions for extracellular HSPs is the modulation of immune activity (Figure 1A and B). For example, secreted HSPA1A induced the production of tumor necrosis factor α (TNFα) and IL-6 in mast cells through the activation of the toll-like receptor 4 (TLR4) and toll-like receptor 2 (TLR2) pathways [111,112,113,114] and the release of interleukin 12 (IL-12) by naive dendritic cells [115]. Additional experiments demonstrated that macrophages infected with bacteria released more HSPA1A containing exosomes and that HSPA1A treatment led to macrophage activation and TNFα release [116]. Tumor cell lines, such as hepatocellular carcinoma cell line HepG2 and murine leukemia monocytes cell lines, were described to secrete HSPD1, HSPA1A, and HSP90AA1 in exosomes, which augmented the cytolytic activity of natural killer cells, macrophages and mononuclear cells [117,118,119,120]. In addition, stimulation of a monocytic cell line with HSPA1A increased cell motility through upregulation of matrix metalloprotease 9 (MMP-9) [121], which could reduce the time necessary for the immune response to infections [122]. Conversely, conflicting results described immunosuppressive functions for HSPA1A. HSPA1A associated with exosomes was shown to reduce tumor immune surveillance by promoting activation of myeloid-derived suppressor cells [123]. However, the data on the function of HSPA1A as a cytokine was challenged by the information of contamination by LPS, as many reports used bacterially derived HSPA1A [124,125,126,127]. To cope with these critics, non-bacterially derived HSPA1A was also used [77,128].

Several other secreted HSPs were linked to immunomodulation (Figure 1). Exogenous HSPD1 treatments induce cytokine release by T cells and macrophages [18]. The secretion of HYOU1 (Grp170), the largest stress protein, stimulates macrophages, which leads to a proinflammatory response that enables pathogen recognition [129]. In cancer, the secretion of HYOU1 enhances immunogenicity and suppresses tumor growth in murine models of melanoma [8] and prostate cancer [7].

Other secreted HSPs may favor an immune escape that presents tumor growth-supporting mechanism (Figure 1B). HSPH1 secreted from colorectal carcinoma cells induced macrophage differentiation favoring a pro-tumor, anti-inflammatory profile [10]. HSPB1 secreted by primary breast tumor cells trigger differentiation of monocytes to macrophages with immunotolerizing phenotypes that lose tumoricidal activity and become proangiogenic [81].

Another proposed function for extracellular HSPs includes modulation of invasiveness and metastasis (Figure 1E). Secretion of HSP90AA1 was implicated in increased cell mobility and cancer invasiveness [3]. HSP90AA1 was first spotted in functional screens for proteins required for the invasion of fibrosarcoma cells [130]. Accordingly, secreted HSP90AA1 promoted breast cancer and melanoma invasion in vitro and increased metastatic potential in animal models. In addition, the presence of serum HSP90AA1 was positively correlated with tumor malignancy in patients presenting cancer of the liver, breast, lung, pancreas or melanoma [131,132]. The inhibition of secreted HSP90AA1 with chemical inhibitors, such as 17-allylamino-17 demethoxygeldanamycin (17AAG) and monoclonal antibody against HSP90AA1 (mAb 4C5), thus reduced in vitro invasion and metastasis in mouse melanoma models [133,134]. The mechanism proposed for the increased invasion is through binding to extracellular receptors, such as the human epidermal growth factor receptor 2 (HER-2), inducing ERK1/2 and PI3K-Akt pathways [135,136]. In non-tumor cell models such as fibroblasts, HSP90AA1 secretion was observed after hypoxia, which triggered increased mobility [137]. Accordingly, motogenic activity of secreted HSP90AA1 was also observed in keratinocytes, triggered by tumor growth factor α (TGFα)-stimulation [138]. Serum starvation also increased HSP90AA1 secretion in colon cancer cells, increasing migration and invasion through NF-κB activation [139].

Other secreted HSPs such as HSPB1 were also linked to increased metastatic properties. In vivo models of murine mammary adenocarcinoma expressing high levels HSPB1 in the cell surface displayed larger tumors when implanted in nude mice, also presenting increased lung metastatic rates [140]. HSPA9 was shown to be secreted by oral squamous carcinoma cells and interact with the adhesion molecule podoplanin, which is involved in cell growth and invasiveness [15].

Secreted HSPs were also linked to angiogenic functions, acting as pro-tumor molecules (Figure 1D). The HSP resident in the ER called HSPA5 (GRP78 or BiP) was observed in cell culture medium and cell membrane after ER stress in human rabdomiosarcoma cells (TE671) and breast cancer cells (MCF7) [141,142] and the ability to secrete HSPA5 has been linked to resistance to the antiangiogenic agent Bortezomib. In angiogenesis experiments, prostate tumor cells (PC-3) and colon (HCT-8) that were able to promote angiogenesis in spite of Bortezomib were shown to secrete HSPA5 and knockdown of this protein abrogated such resistance phenotype [14]. In vivo, antibodies against HSPA5 were found in the circulation of prostate cancer patients [143], and targeting HSPA5 in the surface of tumor cells by the use of chimeric peptides composed of HSPA5 binding motifs fused to a programmed cell death-inducing sequence reduced the tumor growth in preclinical animal models of prostate and breast cancer [144]. Secreted HSPB6 also displayed angiogenic properties. Soluble HSPB6 induced proliferation, migration and tube formation in endothelial cells. In vivo, HSPB6 overexpressing mice also display increased capillary densities in the heart [25]. The angiogenic properties of secreted HSPB1 were related to an ability to cause local differentiation of monocytes into tumor associated macrophages that lose tumoricidal activity but elicit angiogenic responses in breast cancer [23].

In addition to roles in cellular invasion, secreted chaperones were linked to proliferation control (Figure 1C). HSPA8 is secreted in response to high cell density in rat mammary adenocarcinoma cells, inhibiting cell proliferation. Removing HSPA8 from the media by immunodepletion restores proliferation and is associated with the formation of multilayer cell cultures [11].

Additional proposed functions for extracellular HSPs are the transmission of stress resistance, which was demonstrated in neuronal systems. For example, glioma secreted HSPA1A can be up taken by neuroblastoma cells, which increases resistance to induced apoptosis [145]. In cellular and *Drosophila* models, exosomal secretion of DNAJB1 and HSPA1 contributes to the elimination of poly-glutamine aggregates in distant cells [100,146]. Recently, the existence was described of epichaperome machinery, an assembly of multiple chaperone and co-chaperones in cytoplasm, which enables tumor survival [147]. Interestingly, many components of this epichaperome complex were identified as extracellular proteins secreted by exosomes that present biological activity [148,149,150,151,152,153].

## 6. Extracellular HSP-Based Cancer Therapies

The immunomodulatory effects of extracellular HSPs have revealed a potential in the development of cancer therapies. Currently, large classes of chemical inhibitors of intracellular HSPs are under investigation and many are in clinical trials [154,155,156,157]. However, in this review, we will focus only on therapies based on the extracellular activity of HSPs [158].

Examples of anti-tumor activity of secreted HSPs include HSPA1A and HSP90AA1. Heat shock induces the release of exosomes with increased amounts of HSPA1A, which promote antitumor immune responses, with increased expression of major histocompatibility complex (MHC) class II in colon (CT26) and melanoma (B16) models [159], migration and reactivity of natural killer (NK) cells metastatic pancreatic adenocarcinoma Colo357 cells [160], thus inhibiting tumor growth and prolonging survival of tumor-bearing mice (Lewis lung carcinoma cell line and B16 melanoma cell line) [161]. In accordance, when human prostate cell lines overexpressing HSPA1A are injected in mice, there is a significant decrease of tumor growth and increased survival [162]. Similarly, HSP90AA1 and HSPD1 presence in exosomes were related to an increase in immune ability to elicit antitumor immune responses in lymphomas [163].

With the ability of binding to a wide range of extracellular proteins, HSPs are suggested as potential cancer vaccines to control cancer growth. The presence of immunogenic peptides (derived from chaperoned proteins) naturally associated to HSP also reinforces HSP-based vaccines [164]. The antigen-presenting activity of HSP90B1 (Grp96) made this HSP as the prototype for HSP-based vaccines [165]. Clinical studies (from pilot studies to phase I–III trials) were conducted and several are ongoing trying to address the effect of HSP90B1-based vaccines in different tumor types, such as late-stage melanoma [166], metastatic colon carcinoma [167], renal cell carcinoma [168], glioblastoma [169], among others. In general, this type of vaccine is safe, well tolerated, with no toxicity or auto-immune reactions [170]. The outcomes were diverse and clinical response was observed in a limited number of studies [164], such as longer overall survival in stage IV melanoma [171], increased CD8^+^ T cell response in colon cancer patients with metastatic disease [167], reduced tumor-induced lymphopenia and improved survival in glioblastoma patients [169]. An ongoing trial is addressing the effect of vaccination in combination with bevacizumab versus bevacizumab alone in patients with recurrent glioblastoma that underwent surgery (clinical trial number NCT01814813 [172]).

Regarding HSPA1A-based vaccines, a clinical trial was conducted to address the effect of a specific peptide corresponding to a region of HSPA1A, which activates NK cells in refractory metastatic colon cancer and non-small cell lung cancer patients [173]. Peripheral blood lymphocytes from patients were stimulated with the peptide ex vivo and reinfused to address immunological responses. Increased reactivity in NK cells were found together with increased cytolytic activity against HSPA1A-positive tumor targets [173]. Another study in myelogenous leukemia patients in chronic phase addressed the effect of vaccination with patient derived-HSPA1A peptide complexes combined with imatinib mesylate [174]. Clinical responses were observed in 13 of 20 patients, assessed by cytogenetic bone marrow analysis (search for Philadelphia chromosome) and IFN-γ-producing cells with significant correlation between clinical responses and immunological responses when vaccination were combined with imatinib mesylate [174].

## 7. Conclusions

The idea that intracellular chaperones can achieve the extracellular milieu through an active process of cellular secretion was the subject of dispute for decades. However, it is clear today that secretion of HSPs serves many roles and that a number of proteins from this large superfamily are secreted. The mechanisms of secretion are not completely elucidated, even though there is strong evidence for the participation of unconventional secretion, such as exosomes. Especially in the cancer field, the immunomodulatory properties of secreted HSPs may be useful for new vaccine-based therapies and will be the subject of intense exploration in the future.

## Figures and Tables

**Table 1:** Secreted HSPs considered in this review.

Family	HUGO Symbol	Synonyms	Intracellular Function (Gene Cards)	Extracellular Role
HSP70	HYOU1	HSP12A, Grp170	Endoplasmic reticulum (ER)-associated protein involved in stress responses promoted by hypoxia	Mediates cross-presentation in macrophages [6]; enhances immunogenicity [7,8]; potentiates TLR9 activation [9]
	HSPH1	HSP110	Prevents the aggregation of denatured proteins, inhibits HSPA8/HSC70	Binds to scavenger receptors on macrophages and mediates cross-presentation [6]; affects macrophage polarization [10]
	HSPA8	HSC71, HSP73, HSC70	Facilitates peptide folding; ATPase in clathrin-coated vesicle disassembly	Inhibits cell proliferation [11]; dual role in inflammatory response [12,13]
	HSPA1A	HSP70, HSP72	Stabilizes proteins and prevents aggregation; mediates protein folding; involved in the ubiquitin-proteasome pathway	Induces antitumor immune responses [12]
	HSPA5	GRP78, BiP	Involved in the folding and assembly of proteins in the ER	Resistance to antiangiogenic agents [14]
	HSPA9	GRP75	Localized to the mitochondria, ER, and plasma membrane. Role in cell proliferation and stress response	Interacts to adhesion molecule podoplanin and regulates cell growth and metastasis in oral squamous cell carcinoma [15]
Chaperonin	HSPD1	HSP60	Folding and assembly of newly imported proteins in the mitochondria	Tissue regeneration 15[16]; Modulates innate and adaptive immune system 16[17]; Induction of cytokine release [18]
HSPC	HSP90AA1	HSP90, HSP90α	Promotes maturation and structural maintenance of target proteins involved in cell cycle control and signal transduction	Increased in cell mobility and cancer invasiveness; Increase cytokine production, STAT3 activation and MMP9 expression in prostate tumor [19]; Protection against hypoxia via LRP1 [20]
	HSP90B1	GRP94, GP96	Molecular chaperone that functions in the processing and transport of secreted proteins	Antigen-presenting activity [21]
DNAJ	DNAJB1	HSP40	Interacts with HSP70 and stimulates ATPase activity	Binds misfolded protein and inhibits protein aggregation, alleviating toxicity [22]
HSPB	HSPB1	HSP27, Hsp25	Involved in stress resistance and actin organization	Induces macrophage differentiation to M2 [23]; interacts with plasma membrane proteins, altering cell signaling [24]
	HSPB6	HSP20	Heat shock protein that likely plays a role in smooth muscle relaxation	Induces proliferation, migration and tube formation in endothelial cells [25]; Regulator of platelets functions [26]
	CRYAB	HSPB5, αB-crystallin	Hold client proteins in large soluble aggregates; autokinase activity; participation in intracellular architecture	Increased levels associated with photoreceptor neurons death in age-related macular degeneration [27,28]; potential circulating biomarker to predict response to chemotherapy [29]

MMP9, matrix metalloprotase 9; LRP1, Low density lipoprotein receptor-related protein 1; STAT3, Signal transducer and activator of transcription 3; TLR9, Toll-like receptor 9.
